# Endoscopy for Metabolic Diseases

**DOI:** 10.3390/jcm15082832

**Published:** 2026-04-08

**Authors:** Maria Valeria Matteo, Jana Kefah Ibrahim Hussein, Giorgio Carlino, Vincenzo Bove, Valerio Pontecorvi, Loredana Gualtieri, Martina De Siena, Mariachiara Di Vincenzo, Lorenzo Zileri Dal Verme, Daniele Salvi, Clarissa Ferrari, Cristiano Spada, Ivo Boskoski

**Affiliations:** 1 Digestive Endoscopy Unit, Fondazione Policlinico Universitario A. Gemelli IRCCS, 00168 Rome, Italy; mariavaleria.matteo@guest.policlinicogemelli.it (M.V.M.);; 2 Medicine and Surgery Faculty, Università Cattolica del Sacro Cuore, 00168 Rome, Italy; 3 Department of Gastroenterology and Endoscopy, Fondazione Poliambulanza Istituto Ospedaliero, 25124 Brescia, Italy; 4 Research and Clinical Trials Unit, Fondazione Poliambulanza Istituto Ospedaliero, 25124, Brescia, Italy

**Keywords:** endoscopic bariatric metabolic therapies, EBMT, obesity, T2DM, MASLD, metabolic interventions

## Abstract

Endoscopic bariatric and metabolic therapies (EBMTs) offer minimally invasive treatment options for obesity and related metabolic disorders such as type 2 diabetes mellitus (T2DM) and metabolic dysfunction-associated steatotic liver disease (MASLD). These therapies are broadly categorized into gastric and small bowel interventions. Gastric EBMTs, including intragastric balloons and endoscopic sleeve gastroplasty, promote weight loss primarily through mechanical restriction and delayed gastric emptying, thereby improving metabolic outcomes. Small bowel therapies target the proximal intestine to modulate nutrient-sensing and hormonal pathways, providing metabolic benefits that may occur independently of weight loss. Techniques such as duodenal mucosal resurfacing, electroporation-based re-cellularization, and duodenal-jejunal bypass liners demonstrate promising effects on glycemic control, insulin sensitivity, and liver health. Emerging technologies utilizing thermal, vapor, and laser ablation further expand therapeutic possibilities. While these interventions show favorable safety profiles and potential as standalone or adjunctive treatments, further long-term studies and randomized trials are necessary to optimize patient selection and procedural protocols. Collectively, EBMTs represent an evolving paradigm in the management of obesity and metabolic diseases, bridging the gap between conservative medical therapies and bariatric surgery.

## 1. Introduction

Obesity is a chronic, relapsing disorder defined by a body mass index (BMI) of 30 kg/m^2^ or greater and is associated with significant impairment in quality of life as well as increased morbidity and mortality. The global prevalence of obesity has nearly tripled since 1975, establishing it as a major public health challenge [1]. Obesity is closely associated with numerous noncommunicable diseases, including type 2 diabetes mellitus (T2DM), arterial hypertension, cardiovascular disease, metabolic dysfunction-associated steatotic liver disease (MASLD), formerly named nonalcoholic fatty liver disease (NAFLD), musculoskeletal disorders, and certain malignancies [2]. Insulin resistance and compensatory hyperinsulinemia are central to the pathogenesis of these conditions, contributing to metabolic dysregulation and organ dysfunction. T2DM alone is responsible for over one million deaths annually worldwide, underscoring its significant impact on global health [3]. MASLD affects approximately 38% of adults and 7–14% of children and adolescents, with progressive forms such as metabolic-associated steatohepatitis (MASH) resulting from persistent hepatic fat accumulation and inflammation, which may progress to fibrosis and end-stage liver disease. MASLD/MASH is projected to become the leading indication for liver transplantation in the United States [4].

Lifestyle modification, encompassing dietary interventions and increased physical activity, remains the cornerstone of conventional management for obesity, T2DM, and MASLD [5]. Although lifestyle modification can be effective when strictly followed, it often fails to achieve and maintain adequate weight loss. In T2DM and MASLD, clinically meaningful improvements in glycemic control and hepatic parameters typically require a sustained 7–10% reduction in total body weight, a goal reached by only a minority of patients [6]. Pharmacologic therapies have broadened treatment options; modern antidiabetic agents such as glucagon-like peptide-1 receptor agonists (GLP-1RAs) improve glycemic control and, at higher doses, promote weight loss [1]. GLP-1RAs have also shown potential to reverse steatohepatitis, offering both glycemic and hepatic benefits [7]. Recently, a thyroid hormone receptor-β agonist received FDA approval for MASLD, marking the first dedicated pharmacologic therapy for this condition [8]. However, real-world adherence to these therapies remains limited by adverse effects, drug interactions, and the need for therapeutic adherence.

Bariatric surgery, such as Roux-en-Y gastric bypass and sleeve gastrectomy, produces substantial and durable weight loss, ranging from 25% to 30% of baseline weight and significant metabolic improvements [5]. However, its invasive nature, procedural risks, and high costs limit widespread adoption. As a result, fewer than 2% of eligible patients undergo surgery annually [2].

Endoscopic bariatric and metabolic therapies (EBMTs) have emerged as an important bridge between conservative and surgical interventions. EBMTs are broadly categorized based on their primary anatomical target: gastric or small bowel [9]. Gastric interventions primarily promote weight-loss-dependent metabolic improvements, whereas small bowel EBMTs can exert weight-loss-independent effects by modulating key physiological pathways in the duodenum and proximal jejunum. These include alterations in nutrient flow, mucosal remodeling, and neurohormonal reprogramming, which impact glucose metabolism, insulin sensitivity, lipid homeostasis, and hepatic function. Small bowel EBMTs, therefore, exploit the central role of the proximal intestine in the pathogenesis of T2DM and MASLD, offering novel therapeutic avenues for patients who are suboptimally controlled on conventional medical therapy or are unsuitable for surgery [10,11].

Collectively, EBMTs represent a paradigm shift in the management of obesity and metabolic disease, expanding the spectrum of minimally invasive interventions capable of achieving durable metabolic benefits, either through weight-loss-dependent or independent mechanisms, while mitigating the risks associated with traditional bariatric surgery.

## 2. Intragastric Balloons

**Figure 1:**
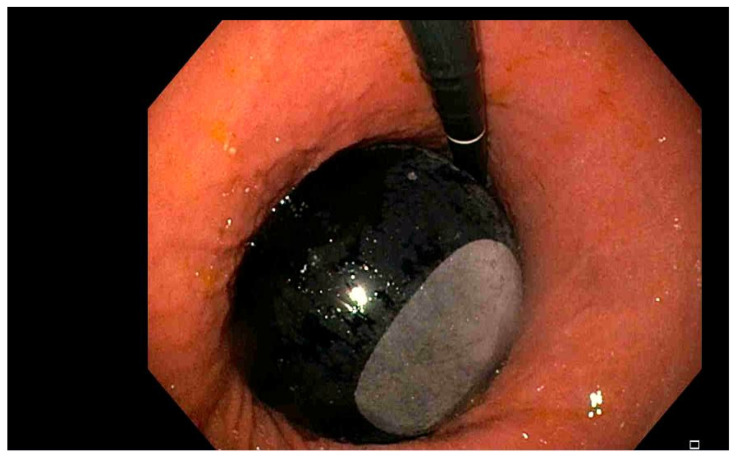
Intragastric balloon (endoscopic view).

Intragastric balloon (IGB) therapy is a minimally invasive, space-occupying endoscopic procedure designed to promote weight loss by reducing the stomach’s functional volume and delaying gastric emptying [9]. During the procedure, one or more silicone balloons are endoscopically introduced into the stomach in a deflated state and then inflated to a volume typically ranging from 400 mL to 700 mL. Inflation is achieved using either a saline solution, often mixed with methylene blue to aid in early leak detection, or, less commonly, a gas [9] (Figure 1). By physically occupying gastric space, IGBs limit the amount of food that can be consumed, prolong gastric retention time, and create a mechanical barrier to intake, thereby enhancing early satiety [12].

Several IGB systems are available, including Orbera™ (Boston Scientific, Marlborough, MA, USA), Spatz3^®^ (Spatz Medical, Great Neck, NY, USA), ReShape™ Duo (Reshape Lifesciences, Irvine, CA, USA), Obalon™ (Obalon Therapeutics, Carlsbad, CA, USA), and Allurion (Allurion Technologies, Natick, MA, USA) [9]. These devices vary slightly in design and filling medium but share the same fundamental mechanism of action [9,11].

Beyond mechanical restriction, IGB therapy appears to induce beneficial neurohormonal changes. Studies have shown that IGB placement may increase secretion of satiety-promoting hormones such as glucagon-like peptide-1 (GLP-1) and peptide YY (PYY), while reducing plasma ghrelin levels, the hormone primarily responsible for stimulating hunger [13,14]. This hormonal modulation—possibly enhanced through vagal signaling—contributes to enhanced satiety, reduced appetite, and decreased caloric intake. The combined mechanical and neurohormonal effects of IGB therapy result in clinically meaningful and sustained weight loss in appropriately selected patients [13,14].

IGBs are commonly employed as a bridging therapy for individuals with severe obesity—particularly those with a very BMI (e.g., >50 kg/m^2^)—to promote preoperative weight reduction and improve the safety and outcomes of subsequent bariatric surgery [5]. Beyond its role as a preparatory measure, IGB therapy has been associated with metabolic improvements, including better glycemic control and favorable changes in liver function parameters. Commercial systems such as the Orbera™ balloon are typically left in place for up to six months to minimize complications such as mucosal injury or ulceration. Removal is generally performed endoscopically [12,15]. Unlike endoscopic gastroplasty procedures, IGB therapy is fully reversible and does not produce permanent anatomical changes in the stomach. However, because the effects are temporary, adherence to dietary and lifestyle modifications after balloon removal is essential to maintain weight loss [5,16].

### 2.1. Efficacy and Weight Loss Outcomes

Evidence from meta-analyses and randomized controlled trials (RCTs) demonstrates that IGB therapy produces clinically meaningful short- to mid-term weight loss. Across studies, the mean total body weight loss (TBWL) at balloon removal (approximately 6 months) ranges from 10.2% to 13.2% [5]. A meta-analysis of 17 studies including 1683 patients using the Orbera™ system reported a mean TBWL of 11.27% at 12 months [17]. Furthermore, RCTs have shown a 6.9% greater TBWL in patients receiving IGB therapy compared with those undergoing lifestyle modification alone at 6–8 months [18]. These findings underscore the efficacy of IGB therapy as a non-surgical approach for achieving clinically relevant weight loss in the short- to intermediate-term.

### 2.2. Metabolic Effects

Given the close relationship between obesity and metabolic dysfunction, the weight reduction achieved through IGB therapy translates into marked improvements in metabolic parameters. A large meta-analysis including 5668 patients across 10 RCTs and 30 observational studies showed that IGB therapy for up to six months resulted in modest but statistically significant improvements in fasting plasma glucose (FPG), HbA1c, systolic blood pressure, and liver transaminases [19]. For instance, HbA1c decreased by −1.1% (95% CI, −0.6 to −1.6), mean FPG decreased by 12.7 mg/dL, with patients with baseline FBG > 100 mg/dL showing an average 15% reduction [19]. Furthermore, the odds ratio for diabetes resolution after IGB therapy was 1.4 (95% CI, 1.3 to 1.6). The odds ratios for hypertension and dyslipidemia resolution were 1.4 (95% CI, 1.3 to 1.6), 2.0 (95% CI, 1.8 to 2.2), and 1.7 (95% CI, 1.2 to 2.6), respectively. Collectively, these findings support the role of IGB as a non-surgical adjunctive treatment for metabolic syndrome and T2DM, with metabolic benefits that extend beyond weight loss.

IGB therapy also confers favorable effects on hepatic steatosis and fibrosis, making it a promising option for patients with MASLD/MASH. Studies have reported significant reductions in hepatic fat content, with the Controlled Attenuation Parameter (CAP) decreasing by an average of −38.7 dB/m after treatment [20]. The NAFLD Activity Score (NAS) improved by about −3 points, and histologic improvement occurred in up to 90% of patients with biopsy-proven MASH [20]. A meta-analysis of nine studies (452 patients) evaluating the Orbera™ system found pooled improvements in NAFLD activity score at histology in 83.5% (95% CI, 60.8 to 94.3) and reductions in steatosis on imaging in 79.2% (95% CI, 66.3–88.1) [21]. These data highlight the potential therapeutic role of IGBs in managing MASLD/MASH. Indeed, the Asian Pacific Association for the Study of the Liver has acknowledged IGBs as a safe and potentially beneficial option for MASLD management. However, evidence remains limited by small patient numbers and short follow-up durations, underscoring the need for further long-term studies—particularly in patients with advanced or end-stage liver disease [6].

### 2.3. Safety

IGB placement is an effective and generally safe endoscopic procedure. The most commonly reported adverse events (AEs) across various IGB systems are nausea (pooled rate 63.3%) and vomiting (55.3%), with the Orbera system showing rates as high as 82% and 72.2%, respectively. Other frequent symptoms include abdominal pain, discomfort, and gastroesophageal reflux [5]. In most cases, these symptoms are transient; however, intolerance may necessitate early balloon removal in up to 5% of patients, with some dual-balloon systems showing early retrieval rates of up to 9% due to nonulcer intolerance [11,12,17]. Severe complications, although uncommon, include mucosal injury or perforation of the stomach or esophagus, gastric ulceration or bleeding, and gastrointestinal obstruction secondary to balloon migration or spontaneous rupture [17,22]. In a large meta-analysis of 801 patients, the rate of serious adverse events (SAEs) was 5.24% (95% CI, 4.84% to 5.64%). Nonetheless, the reported incidence of AEs varies significantly across studies because of several factors, such as differences in sample sizes, variations in pharmacological protocols for antiemetic and pain medications, monitoring frequency of patients, and inconsistencies in the definition of “non-serious” and “serious” AEs [23]. Main contraindications to IGB placement include a history of gastrointestinal surgery, clotting or bleeding disorders, peptic ulcer disease, large hiatal hernia, structural abnormalities of the esophagus or pharynx, and pregnancy [24,25].

## 3. Endoscopic Gastric Remodelling

**Figure 2:**
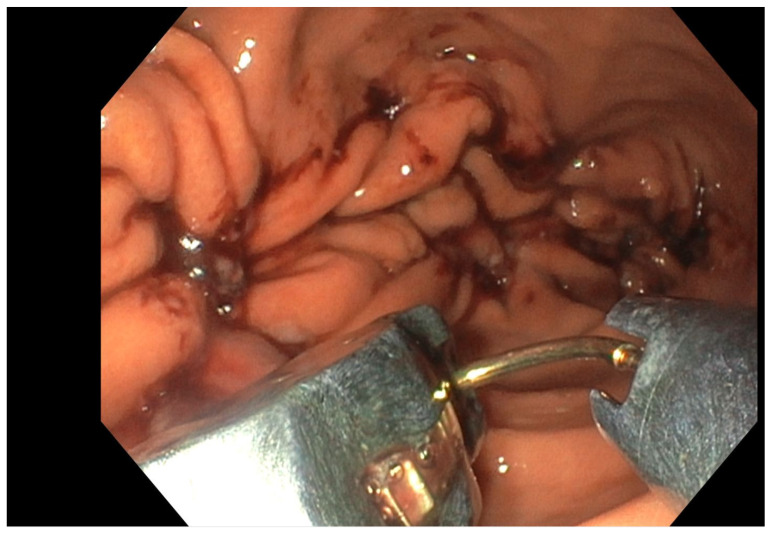
Endoscopic sleeve gastroplasty (endoscopic view).

Endoscopic gastric remodeling (EGR) involves full-thickness suturing of the gastric body, starting at the incisura angularis and extending toward the gastric body, resulting in volume restriction and delayed gastric emptying [9]. Several endoscopic devices are available for EGR, including the Incisionless Operating Platform™ (USGI Medical, San Clemente, CA, USA), the Endomina system (Endo Tools Therapeutics, Gosselies, Belgium), and the EndoZip™ automated suturing system (Nitinotes) [5,11,15]. However, the procedure, also known as Endoscopic Sleeve Gastroplasty (ESG), is most commonly performed using the OverStitch™ Suturing System (Boston Scientific, Marlborough, MA, USA), which is currently the only device with FDA authorization for obesity treatment (Figure 2) [11].

Compared with temporary endoscopic devices such as IGBs, ESG provides greater durability of weight loss outcomes due to its structural remodeling of the stomach [11].

### 3.1. Efficacy and Weight Loss Outcomes

EGR has consistently demonstrated substantial and durable weight-loss outcomes across multiple studies. Meta-analyses report a mean TBWL of approximately 16.5% at 12 months, which remains stable at 17.2% at 18–24 months [26].

The Multicentre ESG Randomised Interventional Trial (MERIT) represents a significant step in demonstrating the effectiveness of ESG. In this study, 209 adults with class I or II obesity were randomly assigned in a 1.5:1 ratio to either undergo ESG with lifestyle changes or to follow lifestyle changes alone [27]. After 52 weeks, the ESG group achieved an average %EWL of 49.2%, whereas the control group achieved only 3.2% (*p* < 0.0001). Additionally, the mean %TBWL was 13.6% in the ESG group versus 0.8% in the control group at the same time point.

Long-term data up to five years post-procedure demonstrate maintained outcomes, with mean TBWL ranging from 14.5% to 15.9%, underscoring the procedure’s durability [28].

### 3.2. Metabolic Effects

EGR has been shown to promote metabolic effects. A prospective single-arm study by Sharaiha et al. reported a significant decrease in HbA1c levels (5.5% ± 0.48 compared to 6.1% ± 1.1, *p* = 0.005), systolic blood pressure (122.2 mmHg ± 11.69 versus 129.0 mmHg ± 13.4, *p* = 0.02), and serum triglycerides (92.36 mmol/dL ± 39.43 compared to 131.84 mmol/dL ± 83.19, *p* = 0.02) at 12 months post-ESG compared to baseline [29].

A large single-arm study by Alqatahni et al. showed remission of hypertension (*n* = 28/28 patients), dyslipidemia (*n* = 18/32 patients), and T2DM (*n* = 13/17 patients) after ESG [30].

Notably, improvements in insulin resistance, as measured by Homeostatic Model Assessment of Insulin Resistance (HOMA-IR), may occur as early as one week after ESG, suggesting early weight-loss-independent metabolic adaptations driven by hormonal and neuroendocrine mechanisms [7].

The multicenter MERIT study has substantiated that patients treated with ESG are more likely than control subjects to demonstrate improvements in T2DM, hypertension, and metabolic syndrome. In a cohort of 91 patients, ESG was associated with significant reductions in HbA1c levels (6.1% to 5.5%), systolic blood pressure (129.0 to 122.2 mmHg), triglycerides (131.8 to 92.4 mg/dL), and alanine aminotransferase (ALT) levels (42.4 to 22.0 U/L in men; 28.0 to 20.0 U/L in women) at 12 months compared to baseline measurements [27].

Furthermore, preliminary evidence indicates beneficial effects of EGR on MASLD/MASH, primarily assessed through non-invasive biomarkers [31,32,33,34]. Jagtap et al. reported a decrease in ALT levels from 59.54 IU/L ± 17.02 at baseline to 48.42 IU/L ± 13.22 at 12 months post-ESG. Furthermore, the surrogate laboratory-based markers, hepatic steatosis index (HSI) and NAFLD fibrosis score (NFS), showed significant improvement after ESG, with HSI decreasing from 44.64 ± 5.19 at baseline to 40.22 ± 4.41 at 6 months and 39.21 ± 4.89 at 12 months, and NFS decreasing from 0.228 ± 1.00 at baseline to −0.202 ± 1.16 at 6 months and −0.552 ± 1.08 at 12 months (*p* = 0.001) [31].

A prospective study of 118 patients reported significant decreases in HSI by average −4 per year) and NFS by −0.3 points per year), with 20% of patients improving fibrosis staging from F3-F4 or indeterminate to F0-F2 [7].

In the study by Jirapinyo et al., involving patients with obesity and underlying MASLD with significant fibrosis (F2–F4), there were notable decreases in ALT (from 49.7 IU/L ± 36.8 to 24.2 IU/L ± 12.0), AST (from 39.1 IU/L ± 24.1 to 24.1 IU/L ± 10.0), NFS (from 0.48 ± 1.51 to −1.18 ± 1.56), and liver stiffness as measured by Fibroscan (from 13.9 kPa ± 7.5 to 8.9 kPa ± 4.8) at 6–12 months [33].

A prospective, non-randomized comparative study (EGR vs. LM) showed a significant decrease in the CAP score, a Fibroscan parameter for hepatic steatosis, in the procedure group from 322.7 dB/m at baseline to 259.5 dB/m and 235.5 dB/m at 6 and 12 months, respectively (*p* < 0.001). In contrast, the control group showed no significant reduction in the mean CAP score [34].

Overall, EGR provides durable weight loss that correlates with significant improvements in non-invasive hepatic steatosis and fibrosis biomarkers, suggesting a beneficial role in managing MASLD. However, data on histological liver outcomes are currently not available. An ongoing randomized controlled trial will clarify the impact of the procedure on biopsy-proven steatohepatitis and fibrosis (NCT04653311).

### 3.3. Safety

EGR techniques have demonstrated a favorable safety profile, with a low rate of SAEs. Reported AEs include perigastric fluid collections, pulmonary embolism, pneumoperitoneum, pneumothorax, post-procedure fever, gastrointestinal bleeding, and severe abdominal pain or nausea requiring hospitalization [35]. Importantly, unlike laparoscopic sleeve gastrectomy (LSG), ESG has not been associated with new-onset gastroesophageal reflux disease (GERD), highlighting a key safety distinction between the two procedures [36].

A meta-analysis of eight observational studies comprising 1772 patients reported TBWL of 15.1% at 6 months, 16.5% at 12 months, and 17.2% at 18–24 months, with a pooled SAE rate of only 2.2% [26]. These events primarily consisted of transient pain or nausea requiring short-term hospitalization, upper gastrointestinal bleeding, or peri-gastric leaks or fluid collections. Moreover, a large propensity score-matched study involving over 3000 patients found that ESG achieved noninferior weight-loss outcomes compared with LSG, with a comparable safety profile [37].

Collectively, EGR offers an effective, minimally invasive, and safe endoscopic alternative to surgical sleeve gastrectomy, combining durable weight loss and metabolic benefits with a low risk of serious complications and minimal procedure-related morbidity.

## 4. Gastric Mucosal Ablation of the Fundus

**Figure 3:**
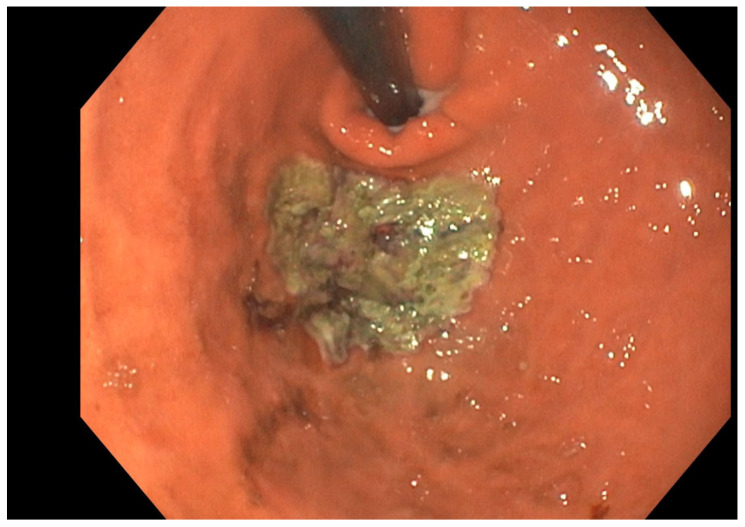
Gastric mucosal ablation of the fundus (endoscopic view).

Endoscopic gastric mucosal ablation (GMA) of the fundus, performed using Hybrid Argon Plasma Coagulation (HybridAPC) probe or MOVIVA probe (ERBE), is an emerging procedure that thermally devitalizes the fundus mucosa to manage obesity by targeting the primary site of ghrelin production [38,39] (Figure 3). By utilizing a submucosal saline cushion to limit the depth of tissue injury, this safe and feasible technique reduces the population of ghrelin-producing cells, leading to a significant 45% decrease in fasting plasma ghrelin levels at six months [40]. This hormonal suppression is particularly promising for metabolic health, as ghrelin is known to promote hyperglycemia and insulin resistance; its reduction may facilitate early glycemic improvement and enhanced insulin sensitivity even before substantial weight loss occurs [41]. Clinical studies have demonstrated that GMA can achieve a mean TBWL of 7.7% as a standalone treatment at six months, while its synergistic application with ESG has shown even greater promise in a pilot trial, reaching a TBWL of 18.64% in the same timeframe [38]. Ultimately, GMA of the fundus represents a potent new tool in the bariatric arsenal, offering a solution for patients who are intolerant to incretin mimetics or who struggle with the compensatory ghrelin spikes that often thwart long-term weight loss [40].

## 5. Small Bowel EBMTs

While most EBMTs primarily achieve their effects through caloric restriction and weight loss, a growing group of small bowel-targeted therapies also provides metabolic benefits that are independent of significant weight reduction, as they directly target metabolic pathways rather than relying solely on fat-mass loss. These interventions focus on the duodenum and proximal jejunum, critical regions for regulating glucose metabolism, insulin sensitivity, and lipid homeostasis [42].

The small intestine plays a central role in the development and progression of metabolic diseases, including T2DM and MASLD. It serves as a key site for nutrient sensing, enteroendocrine hormone secretion, and bile acid signaling, all of which have profound systemic metabolic effects [42]. By modifying nutrient flow, mucosal exposure, and neurohormonal feedback, small bowel endoscopic therapies can enhance directly enhance glycemic and metabolic control even without substantial weight loss.

Bile acid modulation, including sequestration and rerouting, mimics the metabolic effects of surgical bypass and contributes significantly to these outcomes. Altered bile acid flow and enterohepatic circulation enhance fibroblast growth factor 19 (FGF19) and FGF21 signaling, improving hepatic glucose and lipid metabolism [10]. These mechanisms reduce hepatic steatosis and inflammation, highlighting the therapeutic potential of small bowel interventions for MASLD and MASH [10].

Overall, small bowel-focused endoscopic therapies represent a paradigm shift in metabolic intervention. Rather than relying solely on mechanical restriction or malabsorption, they target the underlying pathophysiology of insulin resistance and metabolic dysfunction [42]. By leveraging the endocrine and signaling functions of the proximal intestine, these approaches offer durable metabolic improvements and new therapeutic opportunities for patients with T2DM and MASLD who are not candidates for surgical intervention [43].

### 5.1. Duodenal Mucosal Resurfacing

Duodenal Mucosal Resurfacing (DMR) is a novel endoscopic, catheter-based metabolic intervention designed to improve insulin sensitivity and restore metabolic homeostasis in patients with T2DM and MASLD [44].

The procedure is performed under double endoscopic and fluoroscopic view using the Revita^®^ catheter (Fractyl Health) as a single-session endoscopic intervention consisting of hydrothermal ablation of the duodenal mucosa [1]. Once the duodenum is accessed endoscopically and position of the papilla of Vater is marked with a contralateral endoscopic clip, a submucosal saline injection is performed to create a protective mucosal lift, thereby minimizing thermal injury to deeper layers of the intestinal wall [45]. The submucosal injection is applied longitudinally along the duodenum, extending from approximately 1 cm distal to the ampulla of Vater to the region proximal to the ligament of Treitz [45,46].

Following mucosal lifting, a balloon catheter is inflated with heated water to achieve circumferential thermal ablation of approximately 9–10 cm of the duodenal mucosal surface [44]. Ablation is delivered in multiple, longitudinally spaced applications, typically at temperatures of approximately 90 °C for short durations, with intermittent cooling as required [45]. This controlled thermal injury selectively targets the hypertrophic and metabolically dysregulated duodenal mucosa characteristic of patients with T2DM [2].

After the procedure, patients are generally discharged within 24 h and advised to follow a graduated dietary regimen, progressing from liquids to soft foods over approximately two weeks [1]. Mucosal re-epithelialization occurs rapidly, with functional “resetting” of the duodenal lining observed within days, and complete mucosal regeneration with minimal fibrosis or inflammation is typically evident by three months post-procedure.

The therapeutic rationale for DMR is supported by emerging evidence that the duodenal mucosa in T2DM exhibits aberrant enteroendocrine and absorptive activity [41]. Specifically, overexpression of glucose transporters (SGLT1, GLUT2, GLUT5) and hypersecretion of Glucose-Dependent Insulinotropic Polypeptide (GIP) contribute to excessive insulin output, insulin resistance, and impaired gut–pancreas–liver signaling [46]. By ablating this dysfunctional mucosa and enabling regeneration of a “metabolically healthy” epithelial layer, DMR aims to reset incretin and nutrient-sensing pathways, restore insulin sensitivity, and improve glycemic and hepatic parameters without necessitating substantial weight loss [44]. It is also hypothesized to increase insulin sensitivity, potentially by increasing the production of secondary bile acids [47].

#### 5.1.1. Efficacy

Clinical studies investigating DMR have demonstrated consistent metabolic and hepatic benefits. In the initial proof-of-concept trial, patients with T2DM experienced a mean reduction in HbA1c of approximately 1.2% at 6 months. Reductions in FPG were also observed within 1 week of the procedure, suggesting post-procedural early glycemic effects [48]. Comparative analyses of patients who had received either shorter or longer duodenal ablation lengths demonstrate a length-dependent response, whereby patients undergoing longer segments of duodenal ablation (≥9 cm) exhibited significantly greater reductions in FPG and HbA1c than those treated with shorter ablation lengths (<6 cm) [48]. At three months post-procedure, HbA1c decreased by 27.3 mmol/mol ± 2.2 (2.5% ± 0.2) in the long-ablation group compared with 13.1 mmol/mol ± 5.5 (1.2% ± 0.5) in the short-ablation group (*p* < 0.05) [47]. These findings support a dose–response relationship, with greater glycaemic efficacy associated with longer duodenal mucosal ablation [49].

The multicenter, prospective REVITA-1 study, assessing patients with suboptimally controlled T2DM on oral medications, reported a decrease in HbA1c of −9.8 mmol/mol ± 2.2 (−0.9% ± 0.2) at 24 weeks, along with improved insulin sensitivity, with HOMA-IR showing post-DMR reductions of 2.9 ± 1.1 from baseline [44]. FPG was reduced by 1.7 mmol/L ± 0.5 from a baseline of 10.7 mmol/L ± 2.7 at 6 months (*p* < 0.001). Notably, these improvements were sustained for up to two years, with HbA1c reduced by 0.8% ± 1.3 (*p* = 0.034) and FPG reduced by 34.7 mg/dL ± 36.0 (*p* < 0.001), and occurred independently of weight loss [44]. Indeed, a modest weight reduction of −2.5 ± 0.6 kg (*p* < 0.001) was observed at 24 weeks and −2.4 ± 0.7 kg (*p* < 0.001) at 12 months. This initial weight loss did not show a significant correlation with changes in HbA1c at 24 weeks (Pearson’s correlation 0.29, *p* = 0.14) and 12 months (Pearson’s correlation 0.26, *p* = 0.078), supporting a true metabolic mechanism rather than a weight-mediated effect [44].

The randomized, sham-controlled REVITA-2 trial further confirmed these findings [1]. In the European modified intention-to-treat (mITT) population, patients treated with DMR demonstrated a greater improvement in glycaemic control compared with sham, with a median reduction in HbA1c of –6.6 mmol/mol at 24 weeks, versus –3.3 mmol/mol in the sham group (*p* = 0.033; treatment difference –3.3 mmol/mol). In addition, among European patients with baseline hepatic steatosis (liver MRI-PDFF > 5%), DMR was associated with a median absolute reduction in liver fat content of 5.4% at 12 weeks, accompanied by improvements in hepatic biomarkers, including reductions in ALT levels and FIB-4 fibrosis scores [1]. A recent systematic review corroborated these findings, concluding that DMR significantly improves HbA1c (−0.94%, *p* < 0.001), FPG (−15.84, *p* = 0.020), and hepatic steatosis (liver MRI-PDFF −6.59, *p* < 0.001) at 6 months [50]. Furthermore, a subsequent publication of the REVITA-1 study showed that mean HbA1c significantly decreased from 8.5% at baseline to 7.5% at 6 months and remained stable at 24 months post-DMR, with over half of patients reducing or maintaining glucose-lowering therapy, and ALT levels declined from 38.1 IU ± 21.1 at baseline to 32.5 IU/L ± 22.1 at 24 months (*p* = 0.048) [51].

However, a targeted pilot study by Hadefi et al. evaluating patients with biopsy-proven MASH, characterized by substantially higher baseline liver fat content (23% vs. 8% in REVITA-2), failed to demonstrate MASH resolution or significant metabolic improvements at 12 months in the absence of strong lifestyle interventions [52]. These contrasting findings suggest that while DMR effectively improves glycaemic control and reduces hepatic steatosis in T2DM and early MASLD, its role as a standalone therapy for histological MASH resolution, particularly in patients with more severe disease and advanced fibrosis, remains unproven, a conclusion further supported by recent systematic reviews reporting consistent metabolic and steatosis improvements without definitive histological endpoints.

Taken together, these results support the role of DMR as an effective endoscopic metabolic intervention that improves glycemic control and liver health through duodenal mucosal regeneration and neurohormonal reprogramming, rather than through caloric restriction or malabsorption. As such, DMR represents a promising adjunctive strategy for patients with T2DM and MASLD who remain inadequately controlled on medical therapy or are not candidates for surgical intervention.

Due to its weight loss independent effects on glycemia, a recent trial set out to explore the effect of DMR on euglycemic insulin-resistant states. The DOMINO trial investigated the mechanism of DMR in insulin-resistant women with polycystic ovarian syndrome (PCOS), obesity, and oligomenorrhea [53]. In a double-blind, randomized study of insulin-resistant obese women with PCOS, 32 participants were assigned to either DMR or sham endoscopy. At 12 weeks, whole-body insulin sensitivity, assessed by euglycaemic–hyperinsulinaemic clamp, did not differ significantly between groups, nor did HOMA-IR at 24 weeks, indicating no measurable improvement in insulin sensitivity with DMR in this population [53]. However, from a reproductive standpoint, women undergoing DMR experienced a clinically meaningful increase in menstrual frequency, rising from one cycle in the six months preceding treatment to three cycles in the six months following the procedure, whereas the sham group demonstrated only a modest increase. Although this difference did not reach statistical significance, PCOS-related symptoms improved clinically. These findings suggest that DMR does not uniformly enhance insulin sensitivity across all patient groups and may confer benefit in selected subpopulations. The results further underscore the need for future studies to elucidate the intestinal metabolic mechanisms underlying DMR and to better define patient phenotypes most likely to respond [54].

#### 5.1.2. Safety

DMR is generally well-tolerated, with most adverse events being mild and transient. The most frequent side effects, including abdominal discomfort, transient diarrhea, nausea, vomiting, and occasional asymptomatic hypoglycemia, typically occur within the first few days after the procedure and resolve spontaneously [1].

During the REVITA-1 trial, one SAE was reported that was procedure-related: a transient febrile illness that resolved shortly after [44]. In the REVITA-2 trial, the European population (*N* = 39) did not experience any device-related or procedure-related SAEs through 24 weeks. In the overall safety cohort (DMR *n* = 56), two SAEs (3.6%) related to the procedure were seen in the Brazilian safety population: one precautionary hospitalization for mild hematochezia (possibly related to the procedure) and one jejunal perforation caused by manipulation of the endoscope (requiring surgical repair) [1].

SAEs are uncommon; early reports of duodenal stenosis in initial proof-of-concept studies were successfully managed endoscopically and have been largely mitigated through procedural refinements, including adequate submucosal lifting and avoidance of overlapping ablations [45,48]. Overall, DMR demonstrates a strong safety profile, with adverse effects that are infrequent, manageable, and markedly reduced as the technique has evolved [44].

### 5.2. ReCET

Recellularization via electroporation therapy (ReCET) is a novel, minimally invasive, single-session endoscopic procedure that applies irreversible electroporation (IRE) to the duodenal mucosa via standard upper gastrointestinal endoscopy [55]. The technique uses a dedicated electroporation catheter (Endogenex system) to deliver a pulsed electric field, resulting in controlled ablation of the duodenal mucosa while preserving underlying tissue architecture.

During the procedure, the duodenum is accessed endoscopically, and relevant anatomical landmarks are identified to ensure accurate catheter positioning [9]. Under combined endoscopic and fluoroscopic guidance, the catheter is advanced distally within the duodenum, where the electrode array is deployed. Irreversible electroporation is delivered sequentially in a stepwise fashion, typically treating approximately 10 cm of duodenal length, with each application covering a short axial segment and a substantial proportion of the luminal circumference [9]. The procedure is intended to induce controlled mucosal injury, thereby promoting mucosal regeneration while modulating pathological duodenal signaling pathways implicated in metabolic disease [56].

#### 5.2.1. Efficacy

In the first-in-human study including 14 insulin-dependent diabetic patients, the combination of ReCET with GLP-1Ras therapy led to discontinuation of exogenous insulin in 86% of treated patients [56]. Secondary glycaemic endpoints improved significantly in the overall study population. Median HbA1c decreased from 55 mmol/mol (IQR 53–57) at baseline to 47 mmol/mol (IQR 43–53) at 12 months, while FPG declined from 8.8 mmol/L (IQR 7.6–10.6) to 6.6 mmol/L (IQR 5.8–7.2) [55]. Fasting insulin levels were also significantly reduced, accompanied by a marked improvement in whole-body insulin resistance, with HOMA-IR decreasing from 5.84 (IQR 3.92–7.50) to 1.78 (IQR 1.05–2.71) [56].

No significant changes were observed in microalbuminuria or lipid parameters, including total cholesterol, high-density lipoprotein cholesterol, low-density lipoprotein cholesterol, and triglycerides [56]. However, hepatic steatosis improved substantially, with liver fat fraction decreasing from 9.2% (IQR 6.0–15.4) at baseline to 4.2% (IQR 3.0–10.0) at follow-up [56]. Despite discontinuation of insulin therapy, both glycaemic and metabolic parameters remained significantly improved at 6 and 12 months, with median reductions in HbA1c of 8 mmol/mol and decreases in FPG exceeding 2 mmol/L at 12 months. Overall metabolic outcomes improved markedly, including a median weight loss of 16.7 kg (TBWL 18.4%) at 12 months. Although the authors cannot rule out weight loss as a factor in the glycemic and metabolic benefits, the finding that most patients maintained glycemic control without insulin therapy while on a low dose of GLP1-RA suggests ReCET may have a positive effect on glycemia. Furthermore, approximately 50% reduction in liver fat content was observed after ReCET, supporting the potential role of the procedure as a therapeutic strategy for MASLD [56]. A subsequent publication reporting 24-month follow-up of responders (off insulin and HbA1c ≤ 58 mmol/mol at 12 months) following the ReCET procedure showed that all patients remained off insulin with maintained glycemic control and weight loss (TBWL 19.5%) [56]. Additionally, HOMA-IR and liver fat percentage improved significantly compared with baseline [57].

#### 5.2.2. Safety

The ReCET procedure has demonstrated a favorable safety profile. No procedure-related SAEs have been reported, and follow-up endoscopy revealed normal duodenal mucosa without evidence of scarring [56]. Reported procedure-related adverse events were mild and transient, including sore throat, constipation, and diarrhea, all resolving within a few days. Adverse events related to semaglutide were consistent with its known safety profile, such as nausea and reduced appetite, and no severe hypoglycaemic events were observed [56,57].

### 5.3. Other Systems of Duodenal Ablation

The Radiofrequency Vapor Ablation (RFVA) system (Aqua Medical Inc., Santa Ana, CA, USA) is an emerging endoscopic technology for duodenal mucosal ablation that employs a single-use, through-the-scope catheter [58]. Its through-the-scope delivery obviates the need for fluoroscopic guidance or submucosal lifting, potentially simplifying the procedural workflow relative to over-the-guidewire systems such as Revita^®^ and non-thermal approaches like ReCET. RFVA uses radiofrequency energy to convert saline into high-temperature water vapor (~100 °C) at the distal tip of a bipolar electrode, delivering circumferential thermal energy to the mucosal surface under direct endoscopic visualization without significant injury to deeper layers of the intestinal wall [58]. Vapor is applied in short pulses (typically <5 s per application) to generate focused mucosal coagulation while sparing submucosa and muscularis propria, and sequential ablations are performed in a proximal-to-distal direction targeting a cumulative treated length of ≥9 cm of post-ampullary duodenal mucosa [58]. This controlled thermal injury is intended to induce selective, reversible modification of dysfunctional duodenal mucosa, with subsequent mucosal regeneration and modulation of duodenal nutrient signaling pathways implicated in metabolic disease, similar in principle to other duodenal mucosal ablation techniques. Given the absence of sustained structural damage following RFVA, the metabolic effects are unlikely to result from permanent tissue injury [58]. Instead, it is hypothesized that RFVA modulates the functional interaction between the proximal intestine and luminal glucose, thereby improving downstream hepatic gluconeogenesis and insulin sensitivity. These effects may be mediated through alterations in intestinal glucose sensing, bile acid signaling, local and hepatic gluconeogenesis pathways, or broader transcriptomic changes [58].

Early clinical evidence suggests that RFVA is feasible, safe, and may produce clinically meaningful glycaemic improvements in patients with T2DM. In a first-in-human pilot study, RFVA demonstrated 100% technical success and was well tolerated, with significant reductions in HbA1c and in fasting and post-prandial glucose observed at 12 and 24 weeks post-procedure [58]. No serious adverse events were reported, and follow-up endoscopy showed normal duodenal mucosa without evidence of injury such as strictures or perforation. Preliminary efficacy data indicate mean HbA1c reductions of approximately 1.2% and 0.8% at 12 and 24 weeks, respectively, although full peer-reviewed publication of these results is awaited [58].

The Digma System Endoscopic procedure involves endoscopic deployment of a catheter through the operative channel of a standard endoscope to deliver continuous-wave laser energy (1550–1567 nm) circumferentially to the duodenal submucosa over approximately 24 cm, avoiding the major papilla [59]. The procedure, performed under general anesthesia and lasting about 90 min, uses a balloon to ensure stable contact for precise submucosal laser ablation aimed at modulating duodenal nutrient sensing and metabolic signaling. In a first-in-human, prospective, single-arm study of 31 patients, the procedure demonstrated safety with no device- or procedure-related AEs and showed potential efficacy, including significant reductions in HbA1c at 6 months (−0.6%, *p* = 0.014) and a sustained trend at 12 months (−0.4%, *p* = 0.062), significant FPG reductions at 12 months, improved post-prandial glucose responses, and trends toward reduced insulin resistance, all achieved without significant weight loss, indicating metabolic benefits independent of lifestyle changes [59].

Collectively, these endoscopic duodenal ablation technologies employ distinct energy modalities yet share the common therapeutic objective of selectively modifying pathological duodenal signaling implicated in metabolic disease. While hydrothermal, electroporation-based, vapor-mediated, and laser-based approaches differ in depth of injury, procedural complexity, and extent of duodenal coverage, all aim to induce controlled mucosal or submucosal regeneration, resulting in improved glycaemic control largely independent of weight loss (Table 1). Differences in procedural simplicity, need for fluoroscopy or submucosal lifting, and depth of tissue injury may ultimately influence scalability, safety, and patient selection as these technologies continue to evolve.

## 6. Duodenal-Jejunal Bypass Liner

The Duodenal-Jejunal Bypass Liner (DJBL; RESET, MorphicMedical, Boston, MA, USA), formerly named EndoBarrier (GI Dynamics, Lexington, MA, USA), is a 60-cm-long, self-expanding liner made of fluoropolymer that features a proximal 5.5 cm nitinol self-expandable stent with nitinol barbs [9].

The device is anchored endoscopically in the duodenal bulb, just proximal to the ampulla of Vater, and extends into the proximal jejunum. It functions as a reversible endoluminal bypass, replicating many of the anatomical and hormonal effects of a surgical Roux-en-Y gastric bypass without requiring resection [60].

Once in place, the liner prevents ingested nutrients from contacting the duodenal and proximal jejunal mucosa, altering nutrient absorption and sensing. Pancreatic and biliary secretions flow externally along the sleeve and mix with chyme only at the distal end of the jejunal loop. Beyond weight loss related to reduced nutrient absorption, this bypass effect modulates enteroendocrine signaling, enhancing secretion of incretin hormones such as GLP-1 and PYY while suppressing GIP. Collectively, these hormonal changes improve insulin sensitivity, glycemic control, and lipid metabolism, effectively restoring gut–pancreas–liver communication that is disrupted in metabolic disease [14].

### 6.1. Efficacy

A meta-analysis of 14 studies, including five RCTs (412 patients with T2DM and obesity), DJBL implantation for an average of 8.4 ± 4.0 months resulted in a mean HbA1c reduction of 1.3 percentage points (95% CI, 1.0 to 1.6) and a decrease in HOMA-IR of 4.6 (95% CI, 2.9 to 6.3) [60]. Notably, HbA1c levels remained below baseline by 0.9 percentage points (95% CI, 0.6 to 1.2) 6 months after device removal. Furthermore, TBWL was 18.9% and 7% at explantation and 1 year after removal, respectively [61].

In a prospective cohort of 71 patients with obesity, T2DM, and MASLD treated with DJBL, the Fatty Liver Index decreased significantly from 98.2 at baseline to 93.4 at explantation, further to 90.4 at six-month follow-up (*p* < 0.001) [62]. Additionally, the NFS score improved from 0.19 ± 1.31 at baseline to −0.83 ± 1.4 at device removal (*p* < 0.001), accompanied by a sustained reduction in ALT levels (29.0 vs. 42.3 U/L; *p* < 0.001) at six months post-explantation [62].

A retrospective study analyzing the effects of one-year EndoBarrier treatment on liver fibrosis and steatosis found that among 19 patients, 13 exhibited elevated liver stiffness at baseline consistent with fibrosis grades 2 to 4 [63]. Liver elastography demonstrated a significant reduction in liver stiffness from 10.4 kPa (IQR 6.0–14.3) at baseline to 5.3 kPa (IQR 4.3–7.7, *p* < 0.01) at explantation, indicating normalization of fibrosis in most patients. Although controlled attenuation parameter measurements showed improvement in liver steatosis from 343 dB/m (IQR 326–384) to 317 dB/m (IQR 269–375, *p* < 0.05), most patients remained classified as having high-grade steatosis after treatment.

The ENDOMETAB trial involving 82 patients reported that 12% of DJBL-treated patients achieved remission of metabolic syndrome, compared with 10% of controls [64]. The DJBL group showed significantly greater reductions in BMI (mean adjusted difference −3.1 kg/m^2^; *p* < 0.001) and HbA1c (−0.5%; 95% CI, −0.9 to −0.2; *p* < 0.001) relative to controls; however, these differences were not statistically significant 12 months after device removal [64].

In a multicenter, double-blind, randomized, sham-controlled trial involving 320 subjects (212 DJBL, 108 sham), the DJBL group showed a significantly greater HbA1c reduction at 12 months (−1.10% ± 1.45) compared to sham (−0.28% ± 1.54; *p* = 0.0004). Weight loss was also superior in the DJBL group (7.7% ± 9.6 TBWL vs. 2.1% ± 5.4 TBWL; *p* < 0.0001). More patients in the DJBL arm achieved target HbA1c ≤ 7% (28.3% vs. 9.4; *p* < 0.0003) and TBWL ≥ 5% (60.4% vs. 21.3; *p* < 0.0001) [65].

### 6.2. Safety

Despite its demonstrated efficacy, the DJBL is limited by safety considerations. A systematic review of 1056 patients reported a SAE rate of 3.7%, including 11 hepatic abscesses, 8 gastrointestinal hemorrhages, 4 esophageal perforations, and 3 episodes of acute pancreatitis [66]. The ENDOMETAB trial et al. was halted early due to a 39% incidence of at least one SAE, including liver abscesses. The ENDO trial reported a device-related SAE rate of approximately 9.4%, with common complications including device intolerance (3.7%), gastrointestinal hemorrhage (2.8%), and hepatic abscess formation (2.3%). Recent findings have linked the occurrence of liver abscesses during DJBL therapy to concomitant proton pump inhibitor use [67]. To address safety concerns, the ongoing System Pivotal Trial (STEP-1) is evaluating the device under conditions of restricted proton pump inhibitor administration (NCT04101669).

Reflecting the limited number of patients studied and short-term randomized data, the ASGE–ESGE guideline recommends that DJBL treatment should be performed only within the context of a clinical trial [18].

## 7. Partial Jejunal Diversion with the Incisionless Magnetic Anastomosis System

Partial jejunal diversion (PJD) using the Incisionless Magnetic Anastomosis System (IMAS) is an emerging endoscopic metabolic therapy designed to replicate some of the physiological effects of surgical intestinal bypass in a minimally invasive manner [68]. The technique creates a side-to-side anastomosis between the proximal jejunum and distal ileum, thereby diverting a portion of ingested nutrients away from the mid-small intestine. Technically, the intervention uses two self-assembling octagonal magnets delivered under fluoroscopic guidance—one placed in the jejunum via enteroscopy, the other in the ileum via colonoscopy. Once magnetically coupled, they compress the bowel wall to form a patent anastomosis; after maturation, the magnets pass naturally, leaving a functional enteroenteric connection.

### 7.1. Efficacy

A pilot study showed significant metabolic improvements following the PJD procedure, including a mean HbA1c reduction of 1.9% in patients with type 2 diabetes and a 1.0% decrease in prediabetic individuals, along with marked reductions in post-prandial glucose and insulin levels, indicating enhanced insulin sensitivity. Weight-loss outcomes were notable, with a mean TBWL of 14.6% and an excess weight loss of 40.2% at 12 months. Hepatic function also improved, evidenced by a 23% decrease in ALT levels in diabetic patients, suggesting benefits for MASLD [68].

### 7.2. Safety

Safety data from the pilot study indicated that PJD was generally well tolerated, with mostly mild and transient adverse events such as nausea, diarrhea, and steatorrhea resolving within two weeks. No device-related serious adverse events occurred, and the anastomoses remained patent at one year. A single procedural adverse event involved inadvertent gastric serosal injury during laparoscopic assistance, managed without long-term consequences [68].

## 8. Discussion

EMBTs are minimally invasive procedures that target both gastric and small-bowel anatomy and provide metabolic benefits through distinct mechanisms. A summary of key outcomes across various EBMTs is shown in Table 2.

Gastric therapies primarily induce weight loss via mechanical restriction and delayed gastric emptying, whereas small bowel therapies modulate nutrient sensing and hormonal pathways to achieve weight-loss-independent metabolic benefits. Emerging evidence from clinical studies demonstrates that EBMTs may lead to significant improvements in glycemic control, insulin sensitivity, hepatic function, and weight reduction with favorable safety profiles. Notably, small bowel interventions show promise in modulating enterohepatic signaling and gut hormone secretion, addressing core pathophysiological mechanisms of insulin resistance and metabolic dysfunction. However, the current evidence base is limited by small sample sizes, short- to mid-term follow-up, and the predominance of pilot or first-in-human studies for several small bowel interventions such as ReCET and PJD, which limit the generalizability and robustness of findings. These early-phase studies primarily offer preliminary safety and feasibility data with limited generalizability and statistical power. Although RCTs such as REVITA-1/2 and MERIT provide more robust efficacy and safety data, the overall paucity of large-scale, long-term RCTs hampers definitive conclusions regarding sustained efficacy and safety. This is particularly pertinent given the complex metabolic pathways targeted by these therapies and the need for durable outcomes.

A key limitation across EBMT studies is the lack of histological confirmation of improvement in liver disease in MASLD/MASH, which limits the ability to fully assess the impact of EBMTs on liver pathology and long-term liver-related clinical outcomes. For example, while DMR has demonstrated reductions in hepatic steatosis and improvements in metabolic parameters in a multicenter RCT, it has not consistently shown histological resolution of steatohepatitis, especially in patients with advanced disease. Similarly, weight-loss confounding remains a challenge in interpreting metabolic outcomes, as some therapies, although primarily targeting the small bowel to exert weight-loss-independent effects, may still produce improvements mediated by weight changes. Indeed, in interventions such as ReCET, DJBL, and PJD, although substantial weight loss is observed alongside metabolic improvements, the independent contribution of weight loss versus direct metabolic modulation remains to be fully elucidated. Future studies employing rigorous multivariate adjustments and longitudinal histological assessments are needed to clarify these pathways. The safety profile of EBMTs is generally favorable, though it varies by procedure. IGBs commonly cause transient symptoms such as nausea and vomiting, with SAEs occurring in about 5% of cases. Endoscopic gastric remodeling demonstrates a low SAE rate (<2.2%), primarily involving transient pain or minor bleeding, and notably lacks association with new-onset gastroesophageal reflux disease. Gastric mucosal ablation appears safe with mild, transient effects, while small bowel therapies such as DMR, ReCET, RFVA, and laser ablation report mostly mild, transient AEs and rare SAEs, which have been mitigated through procedural refinements. The DJBL, despite efficacy, carries a higher incidence of serious complications, including hepatic abscesses and gastrointestinal hemorrhages, leading to trial suspensions and restricted use within clinical trials. Partial jejunal diversion is well tolerated, with mild, transient symptoms and no device-related SAEs reported. Overall, while EBMTs offer minimally invasive alternatives with generally low rates of serious complications, uniform safety reporting and long-term data are needed to better define comparative risks and optimize patient selection. Notably, safety data across the studies exhibit considerable heterogeneity, influenced by differences in study design, sample size, definitions of AEs, pharmacologic regimens, and monitoring protocols. For instance, SAEs, though generally infrequent, are variably reported and may be underrepresented in smaller or early-phase studies. Procedural refinements and patient selection criteria remain areas of active investigation to optimize therapeutic outcomes and minimize AEs. With reference to patient selection, according to current ASGE–ESGE guidelines, primary EBMTs are generally indicated for patients with BMI ≥ 30 kg/m^2^, or BMI 27–30 kg/m^2^ with obesity-related comorbidities, particularly when lifestyle and pharmacologic therapies have failed or are not tolerated [18]. Within this framework, gastric therapies are best suited for patients with predominantly obesity-driven disease, especially those with BMI 30–40 kg/m^2^, where weight loss is the primary therapeutic goal. They may also be considered as bridging therapies in patients with BMI > 40–50 kg/m^2^ prior to bariatric surgery, and in individuals with early-stage MASLD, where hepatic improvement is closely linked to weight reduction. In contrast, small bowel-targeted therapies are not yet widely incorporated into formal guidelines and are generally recommended in clinical trials or specialized centers. However, emerging evidence suggests that these interventions may be particularly beneficial in patients with predominant metabolic dysfunction, such as poorly controlled T2DM (e.g., HbA1c ≥ 7.5–8.0%), significant insulin resistance, or MASLD with persistent metabolic activity despite medical therapy, even in those with lower BMI (27–35 kg/m^2^). Overall, a phenotype-based, guideline-informed approach integrating BMI thresholds, T2DM, and MASLD severity may optimize patient selection and clinical outcomes. However, consistent with ASGE–ESGE recommendations, further randomized trials and long-term data are required before expanding indications, particularly for small bowel interventions.

The integration of EBMTs with contemporary pharmacologic agents, including GLP-1RAs and other metabolic drugs, represents a promising avenue for synergistic metabolic control, potentially expanding treatment options for patients inadequately managed by existing therapies. Small bowel EBMTs, such as ReCET, combined with GLP-1RAs have shown potential for insulin discontinuation and sustained metabolic improvement, highlighting opportunities for combined therapies. EBMTs also engage distinct pathways such as neurohormonal modulation and mucosal remodeling, providing durable metabolic benefits independent of weight loss, particularly valuable for patients with advanced metabolic dysfunction or MASLD. However, while GLP-1RAs effectively improve glycemic control and induce weight loss, their real-world use can be limited by AEs, drug interactions, costs, and challenges with patient adherence. In these situations, EBMTs may play a significant therapeutic role. Furthermore, while bariatric surgery remains the most effective option for durable weight loss and metabolic improvement, its use is limited by invasiveness, risks, and low uptake (<2% among eligible patients). Although EBMTs generally yield less weight loss and metabolic effect than surgery, with limited long-term data, their safety, reversibility, and accessibility enhance real-world applicability. In addition to the endoluminal approaches, minimally invasive treatment for obesity includes endovascular bariatric surgery (EBS), which involves selective embolization of the gastric fundus arterial supply, primarily targeting the left gastric artery [69]. This procedure reduces ghrelin production, decreasing appetite and promoting weight loss through hormonal modulation rather than anatomical restriction. EBS has demonstrated promising results for TBWL in small clinical studies, with values ranging from 4.7% to 12.6% at 6–12 months follow-up [69]. While data show promising results following EBS, the technique continues evolving, with studies aimed at optimizing protocols and materials [69]. In the complex scenario of obesity therapy, EBS may serve as an alternative or adjunctive therapy to EBMTs and/or pharmacologic therapies, particularly for patients contraindicated for traditional bariatric interventions. Future research should focus on elucidating the mechanistic underpinnings of these interventions, identifying biomarkers predictive of response, conducting head-to-head comparisons to define the best treatment strategies, and implementing standardized safety assessment and reporting frameworks. Longitudinal studies assessing histological liver outcomes, cardiovascular risk reduction, and quality-of-life measures will be critical for fully characterizing the clinical benefits of EBMTs and other minimally invasive approaches. Additionally, expanding access through streamlined procedural workflows and training will be essential to bridge the current therapeutic gap between medical management and bariatric surgery.

## 9. Conclusions

EBMTs represent a promising advancement in the management of obesity and related metabolic disorders, including T2DM and MASLD. However, given the limited long-term data and modest sample sizes for many technologies, their role should be viewed with caution. As mechanistic understanding, technology, and clinical experience evolve, EBMTs may become integral components of personalized metabolic care, potentially offering safe, effective, and durable alternatives or adjuncts to traditional medical and surgical interventions.

## Figures and Tables

**Table 1:** Characteristics of duodenum-targeted endoscopy devices.

Feature	Revita^®^ (DMR)	ReCET	RFVA	Digma System (Laser)
Energy modality	Hydrothermal (heated water)	Non-thermal Irreversible electroporation	Radiofrequency-generated water vapor	Continuous-wave laser (1550–1567 nm)
Mechanism of tissue injury	Thermal mucosal coagulation	Electrical membrane permeabilization leading to apoptotic cell death	Thermal coagulation via high-temperature vapor (~100 °C)	Irreversible submucosal thermal injury
Catheter type	Over-the-guidewire balloon catheter	Over-the-guidewire electroporation catheter	Through-the-scope catheter	Balloon catheter with rotating laser prism
Need for fluoroscopy	Yes	Yes	No	No
Need for submucosal lifting	Yes	No	No	No
Endoscopic approach	Upper GI endoscopy	Upper GI endoscopy	Upper GI endoscopy	Upper GI endoscopy
Typical treated duodenal length	~9–10 cm post-papillary	~10 cm post-papillary	≥9 cm post- papillary	~24 cm, avoiding the papilla
Primary metabolic effects	↓ HbA1c, ↓ fasting glucose, ↓ liver fat	↓ HbA1c, ↓ insulin resistance, insulin discontinuation	↓ HbA1c, ↓ fasting and post-prandial glucose	↓ HbA1c, ↓ fasting and post-prandial glucose
Effect on liver fat	Significant reductions in selected populations	Marked reductions (≈50%)	Under investigation	N/A
Safety profile	Favorable; rare SAEs	Favorable; no duodenal stenosis/perforation reported	Favorable; mild, transient AEs	Favorable; no major safety signals

DMR: duodenal mucosal resurfacing; N/A: not available; RFVA: Radiofrequency Vapor Ablation; ↓: descrease.

**Table 2:** Summary of EBMTs’ key outcomes.

Procedure	Supporting Evidence	Weight Loss	HbA1c Reduction	Liver Fat/Hepatic Effects	SAE Rate	Regulatory Status
Intragastric Balloons	Metanalysis RCTs Prospective and retrospective non-randomized studies	TBWL 11.27% at 12 months	~1.1% at 6 months	CAP −38.7 dB/m at 6 months NAS −3 at 6 months	5.24%	Orbera™: FDA approved, CE marked Spatz3^®^: FDA approved, CE marked ReShape™ Duo: CE marked Obalon™: FDA approved, CE marked Allurion: FDA approved, CE marked
Endoscopic Gastric Remodelling	Metanalysis RCTs Prospective and retrospective non-randomized studies	TBWL 16.5% at 12 months TBWL 17.2% at 18–24 months TBWL14.5–15.9% at 5 years	~1.5% at 12 months	HSI from 44.64 to 39.21 at 12 months NFS from 0.228 to −0.552 at 12 months LSM from 13.9 kPa to 8.9 kPa at 6–12 months CAP −87.2 dB/m at 12 months	2.2%	OverStitch™: FDA approved, CE marked Endomina^®^: CE marked POSE™: CE marked EndoZip™: CE marked
Gastric mucosal ablation of the fundus	FIH and pilot studies	TBWL 7.7% at 6 months	N/A	N/A	None reported	Hybrid-APC probe: CE-marked MOVIVA probe: CE-marked (Early clinical adoption in specialized centers)
Duodenal Mucosal Resurfacing (Revita)	Metanalysis RCTs Single-arm prospective studies	AWL −2.4 kg ± 0.7 at 6 months	~1% at 6–24 months	~−1.0% up to 24 months	~2–3%	CE-marked (commercially available in Germany and UK)
ReCET	FIH study	TBWL 18.4% at 12 months TBWL 19.5% at 24 months	~0.7% at 12–24 months	~50% reduction in liver fat at 2 months	None reported	Investigational
RF Vapor Ablation	FIH study	AWL–2.4 kg ± 5.0,at 12 weeks AWL–1.2 kg ± 5.2 at 24 weeks	0.8% at 12 weeks 1.2% at 24 weeks	N/A	None reported	Investigational
Digma Laser	FIH study	Minimal variation (not significant)	0.4% at 6 months 0.6% at 12 months	N/A	None reported	Investigational
Duodenal-Jejunal Bypass Liner	Metanalysis RCTs Prospective and retrospective non-randomized studies	TBWL 18.9% at 8.4 ± 4.0 months TBWL 7% at 12 months after explantation	1.3% at 8.4 ± 4.0 months 0.9% at 6 months after explantation	NFS from 0.19 ± 1.31 to −0.83 ± 1.4 at explantation LSM from 10.4 kPa to 5.3 kPa at explantation	3.7%	Investigational (CE mark revoked due to safety issues)
Partial Jejunal Diversion	FIH study	14.6% at 12 months	1.9% in T2DM patients at 12 months 1.0% in prediabetic patients at 12 months	ALT reduction 23% at 12 months	None reported	Investigational

ALT: alanine liver transaminase; APC: argon plasma coagulation; AWL: absolute weight loss; CAP: controlled attenuation parameter; FIH: first in human; LSM: liver stiffness measurement; HSI: hepatic steatosis index; N/A: not available; NAS: NAFLD activity score; NFS: NAFLD fibrosis score; RCT: randomized controlled study; T2DM: type 2 diabetes mellitus; TBWL: total body weight loss.

## Data Availability

No new data were created or analyzed in this study.
